# Dynamic Modeling for Chatter Analysis in Micro-Milling by Integrating Effects of Centrifugal Force, Gyroscopic Moment, and Tool Runout

**DOI:** 10.3390/mi15020244

**Published:** 2024-02-06

**Authors:** Xiaoli Liu, Dexuan Liu, Canyang Du, Yang Li, Caidong Wang, Zhijun Fu

**Affiliations:** 1 College of Mechanical and Electrical Engineering, Zhengzhou University of Light Industry, Zhengzhou 450002, China; liuxiaolizzuli2018@163.com (X.L.); dexuanliu21@163.com (D.L.); ducanyang123@163.com (C.D.); yanglizzuli@163.com (Y.L.); fuzhijun2008@163.com (Z.F.); 2 Henan Provincial Key Laboratory of Intelligent Manufacturing of Mechanical Equipment, Zhengzhou 450002, China

**Keywords:** regenerative chatter, centrifugal force, gyroscopic effect, tool runout, micro-milling process

## Abstract

During micro-milling, regenerative chatter will decrease the machining accuracy, destabilize the micro-milling process, shorten the life of the micro-mill, and increase machining failures. Establishing a mathematical model of chatter vibration is essential to suppressing the adverse impact of chatter. The mathematical model must include the dynamic motions of the cutting system with the spindle–holder–tool assembly and tool runout. In this study, an integrated model was developed by considering the centrifugal force induced by rotational speeds, the gyroscopic effect introduced by high speeds, and the tool runout caused by uncertain factors. The tool-tip frequency-response functions (FRFs) obtained by theoretical calculations and the results predicted by simulation experiments were compared to verify the developed model. And stability lobe diagrams (SLDs) and time-domain responses are depicted and analyzed. Furthermore, experiments on tool-tip FRFs and micro-milling were conducted. The results validate the effectiveness of the integrated model, which can calculate the tool-tip FRFs, SLDs, and time responses to analyze chatter stability by considering the centrifugal force, gyroscopic effect, and tool runout.

## 1. Introduction

Microproducts are characterized by small sizes, complex structures, and high precision. They are used in the aerospace, biomedical, optical, and electronics fields. They can be manufactured by micro-milling with a tool diameter of less than 1 mm [1].

During machining, regenerative chatter vibration is self-excited by the cutting process, decreasing the surface precision and causing the instability of the micro-milling process while shortening the life of the micro-mill and increasing the probability of machining failure [2]. Regenerative chatter vibration mainly refers to vibrations between the cutting tool and the workpiece. During micro-milling, the machined surface of the workpiece produced by the previous cutting tooth is the surface machined by the next cutting tooth, indicating a time delay between two successive cutting processes. An unexpected resonance between the cutting force and a specific mode of the machining system may occur. In such instances, the previous tooth may leave ripples on the surface of the workpiece, and the subsequent tooth may produce new ripples, providing a phase difference in the machined chip thickness. Consequently, the chip thickness variation will cause dynamic fluctuations in the corresponding cutting forces. In addition, the variable cutting forces will act on the relative cutting between the tool and the workpiece. Hence, the formation of the chip thickness is affected. If such cutting processes occur repeatedly, they will cause an increase in the chip thickness and cutting forces, leading to regenerative chatter and ultimately destabilizing the cutting system.

Developing an analytical model that includes key influencing factors is crucial to demonstrating the influences of chatter. The main influencing factors must include the dynamic motions of the spindle–holder–tool assembly. During cutting, the spindle speed of the micro-machine is usually higher than 10,000 revolutions per minute to ensure a high cutting efficiency in micro-milling [3]. Therefore, the centrifugal force induced by rotational speeds and the gyroscopic effect induced by high speeds are inevitably accompanied by high spindle speeds. Wang et al. [4] constructed a mathematical model for the spindle system. The authors used the Timoshenko beam element and considered the effects of beam bending, transverse shear, and gyroscopic moments. Furthermore, the model can be employed to perform a dynamic analysis. Feng et al. [5] discretized the spindle into classical Timoshenko beam elements, simplified the rotor as a rigid disk, and developed the mathematical model of the spindle system by incorporating the centrifugal force and gyroscopic moment. The authors developed a spindle-bearing dynamic model for vibration-response analysis. Lu et al. [6] developed a model of the spindle–tool assembly by employing the centrifugal force and gyroscopic moment. The model can be used to simulate and predict chatter stability. Shi et al. [7] constructed a mathematical model of the spindle system considering the effects of centrifugal force, gyroscopic moments, and bearing coupling by employing the Timoshenko beam element and shaft tilt deformation. The model can be applied to investigate chatter stability. Hentati et al. [8] employed Timoshenko beam elements with distinct circular sections to equalize the spindle and demonstrate the dynamics of spindle-rolling bearing systems for chatter analysis. The authors incorporated the gyroscopic effect and centrifugal force. Lee et al. [9] employed a Timoshenko beam element to construct the spindle system by considering the influences of eccentric mass and the gyroscopic effect. The established model can predict spindle system vibration. The models mentioned above include the effects of gyroscopic moments, centrifugal forces, or both, without the influence of tool runout.

However, including the effects of tool runout during the dynamic modeling of chatter vibration is crucial because the tool runout caused by installation errors, manufacturing errors, and cutting uncertainties will change the tool point’s nonlinear trajectory and cutting forces. Consequently, the surface precision will be decreased at the micro-scale during micro-milling, affecting chatter stability. Zhang et al. [10] obtained cutting forces with the effects of the micro-size and tool runout. Wimmer et al. [11] described the effects of tool runout using the radial tool runout and jump angle. The authors incorporated the effects into the instantaneous uncut chip thickness and applied the thickness to construct the expression of cutting forces. Totis et al. [12] established an improved model to describe the milling forces. The established model contains the influence of tool runout, geometry, and forced vibration on the effective engagement condition of the tool and workpiece. Wang et al. [13] obtained the milling force model by combining the tool eccentricity distance with the runout angle. The papers mentioned above mainly focus on modeling the cutting forces with tool runout; as such, they do not involve the centrifugal force and gyroscopic effect.

Researchers have tried to improve the mathematical model of spindle–holder–tool systems by considering the centrifugal force and gyroscopic effect. Furthermore, several investigations have been performed to model the cutting forces with tool runout. However, the works mentioned above need to be further developed to obtain comprehensive modeling for describing the incorporation of the centrifugal force, gyroscopic effect, and tool runout of chatter vibration in micro-milling.

A dynamic model incorporating the centrifugal force, gyroscopic effect, and tool runout into the motion of spindle–holder–tool assembly is presented in this paper by following previous investigations [6,7,14,15]. In this paper, the introduction is presented in Section 1, which describes the need for comprehensive approaches to modeling the effects of the centrifugal force, gyroscopic moment, and tool runout of chatter vibration in micro-milling. According to this demand, a mathematical model has been developed by using the finite element method and time-domain modeling and is illustrated in Section 2, Section 3 and Section 4. In Section 2, the motion equations for a beam unit with the centrifugal force and the gyroscopic effect are illustrated. Then, the equations of cutting forces considering the tool runout distance and the runout angle are described in Section 3. Subsequently, the assembling of the equations in Section 2 and Section 3 is expressed in Section 4. Following the assembled equation, it is verified in Section 5 by analyzing the experimental and predicted tool-tip FRFs. Based on the FRFs, the SLDs are described in Section 6. According to the results of SLDs, an analysis of the time responses of vibrations is demonstrated in Section 7. Finally, the conclusions are drawn in Section 8.

## 2. The Kinetic Equations of a Beam Unit with Centrifugal Force and the Gyroscopic Effect

**Figure 1:**
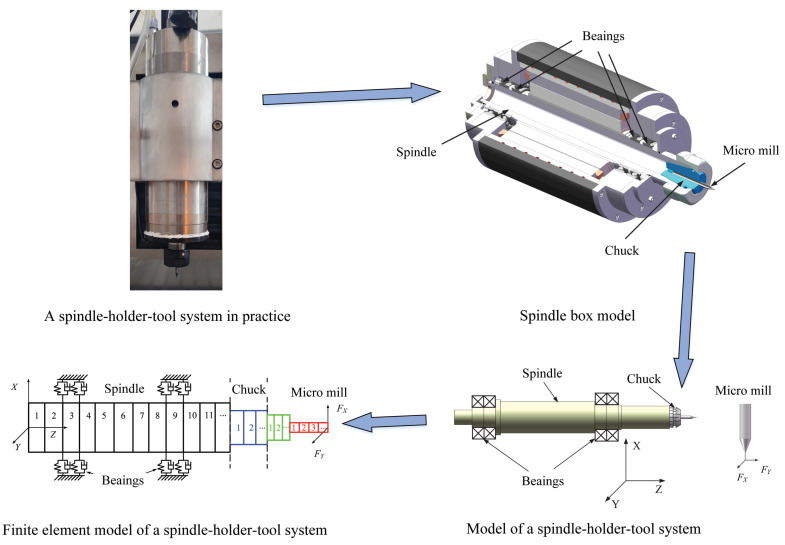
A spindle–holder–tool system.

Figure 1 illustrates a practical spindle–holder–tool assembly, which includes a spindle, holder, and tool. The effects of the holder and spindle cannot be neglected when investigating the dynamics of a rotating micro-mill because they provide the driving torque and clamping action, respectively. The micro-mill could not rotate without them, losing its function as a tool.

As illustrated in Figure 1, the spindle–holder–tool system can be divided into four parts according to the design size and configuration assembly [9,16,17]. If each part is divided into several segments, the assembly can be simplified [5,7,18].

### 2.1. Kinetic Energy, Potential Energy, and Work Performed by External Forces for a Rotating Beam Unit

**Figure 2:**
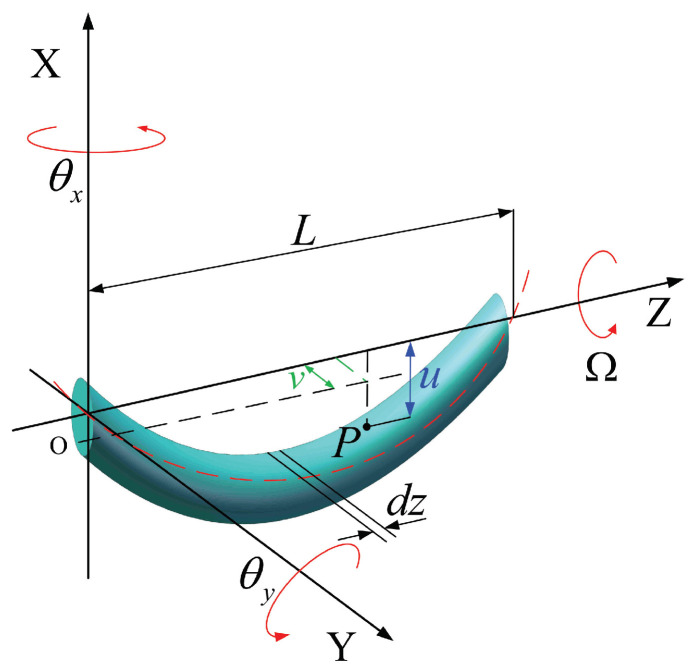
Timoshenko element.

The Timoshenko beam shown in Figure 2 is employed to model the segment with beam bending. The length of the rotating Timoshenko beam is *L*. The kinetic energy can be derived as follows [19]:

(1) $$\left\{\begin{array}{c} T\;=\;T_{1}+T_{2}+T_{3}\\ T_{1}\;=\;\frac{1}{2}\int_{0}^{L}\rho J\Omega^{2}dz\\ T_{2}\;=\;\frac{1}{2}\int_{0}^{L}\rho A\left({\dot{u}}^{2}+{\dot{v}}^{2}\right)dz\\ T_{3}\;=\;\frac{1}{2}\int_{0}^{L}\rho I\left({\dot{\theta }}_{x}^{2}+{\dot{\theta }}_{y}^{2}\right)dz \end{array}\right.$$

where $T_{1}$ represents the polar area effect of rotatory inertia, $T_{2}$ represents the translational energy, and $T_{3}$ expresses the motion for beam bending. The displacements and rotations of point *P* on the beam unit are denoted by ${\left\{\begin{array}{cccc} u & v & \theta_{x} & \theta_{y} \end{array}\right\}}^{T}$, the velocities are expressed by ${\left\{\begin{array}{cccc} \dot{u} & \dot{v} & {\dot{\theta }}_{x} & {\dot{\theta }}_{y} \end{array}\right\}}^{T}$, Ω denotes the rotational speed, ρ is the material’s density, *A* is the cross-sectional area, $J\;=\;0.5\pi R^{4},I\;=\;0.25\pi R^{4}$, and *R* denotes the radius of the beam section.

The potential energy of a beam element is given as [20]

(2) $$\left\{\begin{array}{c} V\;=\;V_{1}+V_{2}\\ V_{1}\;=\;\int_{0}^{L}\frac{1}{2}K_{S}AG\gamma^{2}dz\\ V_{2}\;=\;\frac{1}{2}EI\int_{0}^{L}{\left(\frac{d\theta }{dz}\right)}^{2}dz \end{array}\right.$$

where $V_{1}$ represents the motion equation for the shear strain energy, $V_{2}$ indicates the motion equation for the bending strain energy, $K_{s}$ is the shear coefficient, γ is the shear deformation angle, *G* represents the shear modulus, and *E* denotes the modulus of elasticity.

**Figure 3:**
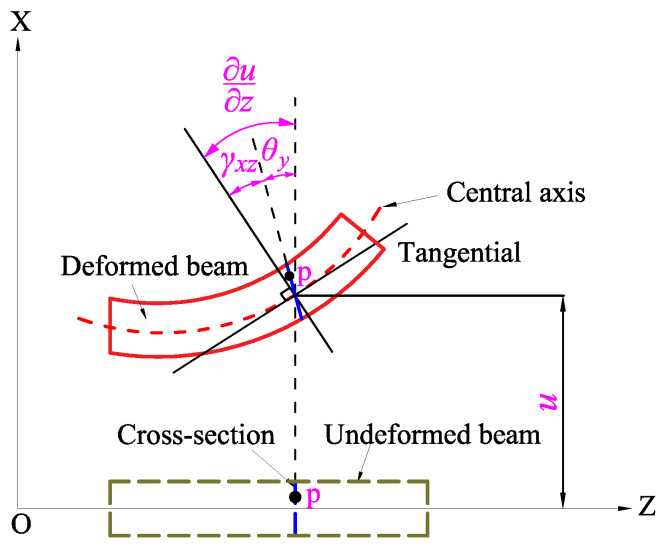
The original shape and deformation of a beam in the X-Z plane.

As shown in Figure 3, the shear deformation angles of the beam unit due to bending are obtained as follows [21]:

(3) $$\gamma_{xz}\;=\;\frac{\partial u}{\partial z}-\theta_{y}$$

and

(4) $$\gamma_{yz}\;=\;\frac{\partial v}{\partial z}-\theta_{x}$$

The potential energy can be rewritten by substituting Equations (3) and (4) into Equation (2):

(5) $$\left\{\begin{array}{c} V\;=\;V_{1}+V_{2}\\ V_{1}=\frac{1}{2}K_{s}AG\int_{0}^{L}\left[{\left(\frac{\partial u}{\partial z}-\theta_{y}\right)}^{2}+{\left(\frac{\partial v}{\partial z}+\theta_{x}\right)}^{2}\right]dz\\ V_{2}=\frac{1}{2}EI\int_{0}^{L}\left[{\left(\frac{\partial \theta_{x}}{\partial z}\right)}^{2}+{\left(\frac{\partial \theta_{y}}{\partial z}\right)}^{2}\right]dz \end{array}\right.$$

The work performed by external forces and torques is expressed by [22]

(6) $$W_{1}\;=\;\int_{0}^{L}\left(F_{x}u+F_{y}v+M_{x}\theta_{x}+M_{y}\theta_{y}\right)dz$$

where $F_{x}$ and $M_{x}$ represent the external force and external moment in the X-direction, respectively, while $F_{y}$ and $M_{y}$ represent the external force and external moment in the Y-direction, respectively.

### 2.2. Equations of External Work by Incorporating Centrifugal Force

**Figure 4:**
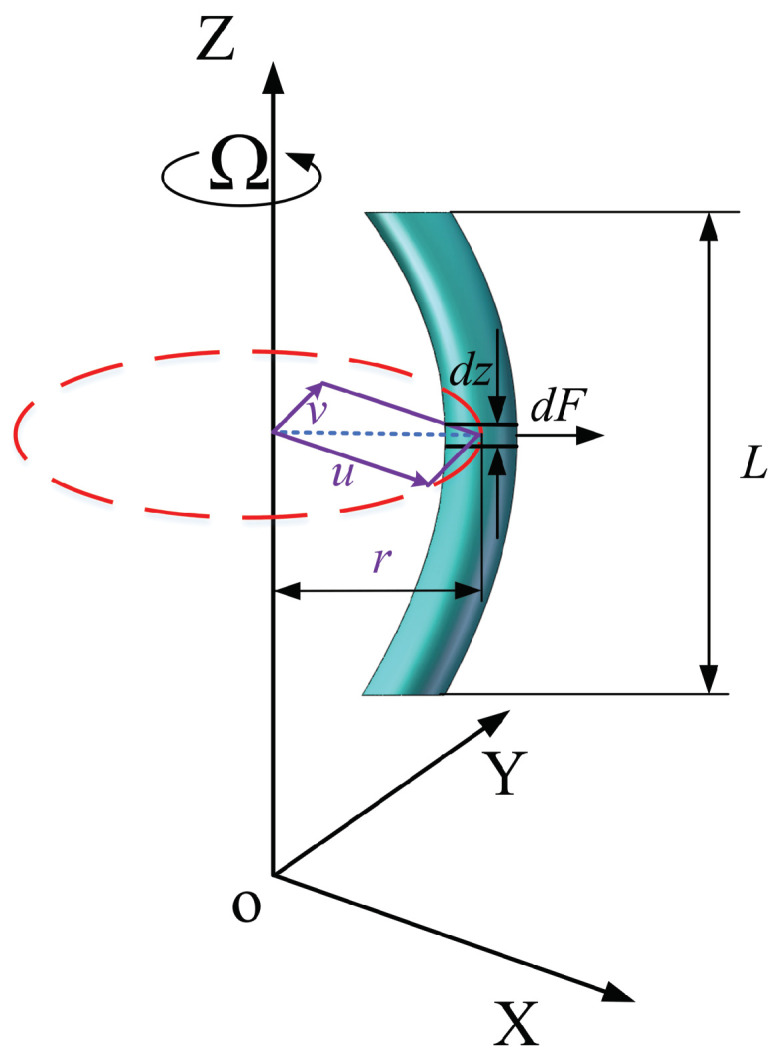
Rotating beam with centrifugal force.

As demonstrated in Figure 4, the centrifugal force acting on a segment of a rotating beam is given as follows:

(7) $$dF\;=\;\rho Ar\Omega^{2}dz$$

where *r* denotes the radius of rotation, and dz indicates the element length.

The external virtual work is obtained as follows [23]:

(8) $$dW\;=\;\frac{1}{2}dFr\;=\;\frac{1}{2}\rho Ar^{2}\Omega^{2}dz$$

Furthermore, integrating the expression dW for a beam segment with length *L* yields [23]

(9) $$W_{2}\;=\;\int_{0}^{L}\frac{1}{2}\rho A\Omega^{2}r^{2}dz\;=\;\int_{0}^{L}\frac{1}{2}\rho A\Omega^{2}u^{2}dz+\int_{0}^{L}\frac{1}{2}\rho A\Omega^{2}v^{2}dz$$

### 2.3. Equation of Kinetic Energy by Including Gyroscopic Effect

**Figure 5:**
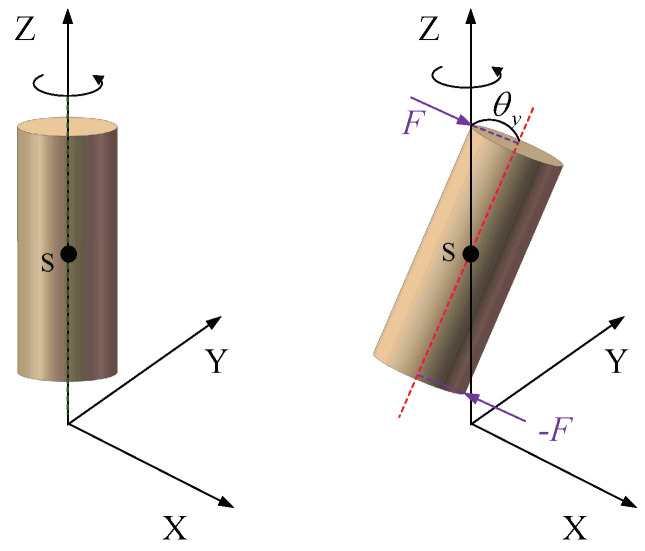
Effect of gyroscopic moment.

In Figure 5, the moment of inertia about the *Z*-axis is $J_{z}$, and the beam unit centroid is *S*. The gyroscopic moment about the *Z*-axis in the *Y*-direction can be expressed as follows [24]:

(10) $$M_{ty}\;=\;-J_{z}\Omega {\dot{\theta }}_{y}$$

And the gyroscopic moment about the Z-axis in the X-direction can be expressed as

(11) $$M_{tx}\;=\;-J_{z}\Omega {\dot{\theta }}_{x}$$

Summing the above two equations yields the kinetic energy generated by the gyroscopic moments acting on the beam unit [4,14]:

(12) $$T_{4}\;=\;\frac{1}{2}\int_{0}^{L}\rho J\Omega \left({\dot{\theta }}_{x}\theta_{y}-{\dot{\theta }}_{y}\theta_{x}\right)dz$$

### 2.4. Finite Element Equations

The dynamic modeling of the spindle–holder–tool system incorporates the effect of gyroscopic moments. Additionally, dynamic modeling includes the influence of the centrifugal force. The equation describing the total work performed by external forces and torques, as well as the centrifugal force, will be derived by summing Equations (1) and (12) as follows:

(13) $$T\;=\;\frac{1}{2}\int_{0}^{L}\rho J\Omega^{2}dz+\frac{1}{2}\int_{0}^{L}\rho A\left({\dot{u}}^{2}+{\dot{v}}^{2}\right)dz+\frac{1}{2}\int_{0}^{L}\rho I\left({\dot{\theta }}_{x}^{2}+{\dot{\theta }}_{y}^{2}\right)dz+\frac{1}{2}\int_{0}^{L}\rho J\Omega \left({\dot{\theta }}_{x}\theta_{y}-{\dot{\theta }}_{y}\theta_{x}\right)dz$$

The potential energy can be obtained by summing the formulas in Equation (5):

(14) $$V\;=\;\frac{1}{2}EI\int_{0}^{L}\left[{\left(\frac{\partial \theta_{x}}{\partial z}\right)}^{2}+{\left(\frac{\partial \theta_{y}}{\partial z}\right)}^{2}\right]dz+\frac{1}{2}K_{s}AG\int_{0}^{L}\left[{\left(\frac{\partial u}{\partial z}-\theta_{y}\right)}^{2}+{\left(\frac{\partial v}{\partial z}+\theta_{x}\right)}^{2}\right]dz$$

The work performed by external forces, torques, and the centrifugal force is formulated by adding Equations (6) and (9):

(15) $$W\;=\;\int_{0}^{L}\left(F_{x}u+F_{y}v+M_{x}\theta_{x}+M_{y}\theta_{y}\right)dz+\int_{0}^{L}\frac{1}{2}\rho A\Omega^{2}u^{2}dz+\int_{0}^{L}\frac{1}{2}\rho A\Omega^{2}v^{2}dz$$

Rewriting Equations (13)–(15) results in

(16) $$T\;=\;\int_{0}^{L}\frac{1}{2}J\rho \Omega^{2}dz+\frac{1}{2}{\left\{\dot{q}\right\}}^{T}\left[M^{e}\right]\left\{\dot{q}\right\}+\frac{1}{2}\Omega {\left\{\dot{q}\right\}}^{T}\left[G^{e}\right]\left\{q\right\}$$

(17) $$V\;=\;\frac{1}{2}{\left\{q\right\}}^{T}\left[K^{e}\right]\left\{q\right\}$$

(18) $$W\;=\;\left[F^{e}\right]\left\{q\right\}+\frac{1}{2}\Omega^{2}{\left\{q\right\}}^{T}\left[{M_{c}}^{e}\right]\left\{q\right\}$$

with

$$\left[M^{e}\right]\;=\;\int_{0}^{L}{\left[N\right]}^{T}\left[\begin{array}{cccc} \rho A & 0 & 0 & 0\\ 0 & \rho A & 0 & 0\\ 0 & 0 & \rho I & 0\\ 0 & 0 & 0 & \rho I \end{array}\right]\left[N\right]dz\;\left[G^{e}\right]\;=\;\int_{0}^{L}{\left[N\right]}^{T}\left[\begin{array}{cccc} 0 & 0 & 0 & 0\\ 0 & 0 & 0 & 0\\ 0 & 0 & 0 & J\rho \\ 0 & 0 & -J\rho & 0 \end{array}\right]\left[N\right]dz$$

$$\left[K^{e}\right]\;=\;\int_{0}^{L}{\left[\begin{array}{c} \left[{N^{\prime }}_{\theta x}\right]\\ \left[{N^{\prime }}_{\theta y}\right]\\ \left[N_{\theta x}\right]+\left[{N^{\prime }}_{v}\right]\\ \left[N_{\theta y}\right]-\left[{N^{\prime }}_{u}\right] \end{array}\right]}^{T}\left[\begin{array}{cccc} EI & 0 & 0 & 0\\ 0 & EI & 0 & 0\\ 0 & 0 & K_{s}AG & 0\\ 0 & 0 & 0 & K_{s}AG \end{array}\right]\left[\begin{array}{c} \left[{N^{\prime }}_{\theta x}\right]\\ \left[{N^{\prime }}_{\theta y}\right]\\ \left[N_{\theta x}\right]+\left[{N^{\prime }}_{v}\right]\\ \left[N_{\theta y}\right]-\left[{N^{\prime }}_{u}\right] \end{array}\right]dz$$

$$\left[F^{e}\right]\;=\;\int_{0}^{L}{\left[\begin{array}{c} f_{x}\\ f_{y}\\ m_{x}\\ m_{y} \end{array}\right]}^{T}\left[N\right]dz\;\left[M_{c}^{e}\right]\;=\;\int_{0}^{L}{\left[N\right]}^{T}\left[\begin{array}{cccc} \rho A & 0 & 0 & 0\\ 0 & \rho A & 0 & 0\\ 0 & 0 & 0 & 0\\ 0 & 0 & 0 & 0 \end{array}\right]\left[N\right]dz$$

where ${\left\{\begin{array}{cccc} u & v & \theta_{x} & \theta_{y} \end{array}\right\}}^{T}=\left[N\right]\left\{q\right\}$, *q* represents the displacements and rotations of the nodes at both ends of the beam, $\left[N\right]={\left[N_{u},N_{v},N_{\theta x},N_{\theta y}\right]}^{T}$ is the shape function, $\left[M^{e}\right]$ represents the mass matrix of a beam unit, $\left[G^{e}\right]$ denotes the gyroscopic matrix, $\left[K^{e}\right]$ denotes the stiffness matrix, $\left[M_{c}^{e}\right]$ denotes the matrix utilized to reflect the centrifugal force effect, and $\left[F^{e}\right]$ denotes the force vector representing external forces.

The following equation can be obtained according to Hamilton’s Principle and the Law of Conservation of Energy [25]:

(19) $$\delta \int_{t_{1}}^{t_{2}}\left(T-V+W\right)dt\;=\;0$$

Substituting Equations (16)–(18) into Equation (19) yields

(20) $$\left[M^{e}\right]\left\{\ddot{q}\right\}-\Omega \left[G^{e}\right]\left\{\dot{q}\right\}+\left(\left[K^{e}\right]-\Omega^{2}\left[M_{c}^{e}\right]\right)\left\{q\right\}\;=\;\left[F^{e}\right]$$

## 3. Equations of Cutting Forces Considering Tool Runout Distance and Runout Angle

In fact, tool runout may occur in the radial and axial directions due to the complex cutting conditions during micro-milling. The tool runout with the runout distance and runout angle in radial directions has been one of the research hotspots in recent years [10,12,13], without considering the tool runout in the axial direction. Moreover, the regenerative chatter in radial directions has been investigated without considering the vibration in the axial direction in recent research [7,8]. This may be because the tool stiffness in the axial direction is usually sufficiently large to surpass unexpected vibrations during cutting due to the large length-to-diameter ratio of the tool. In this paper, the tool runout in radial directions is investigated without considering the tool runout in the axial direction.

Models of the cutting forces, including tool runout, are established based on the actual trajectory of the micro-mill with runout due to uncertainties during cutting.

**Figure 6:**
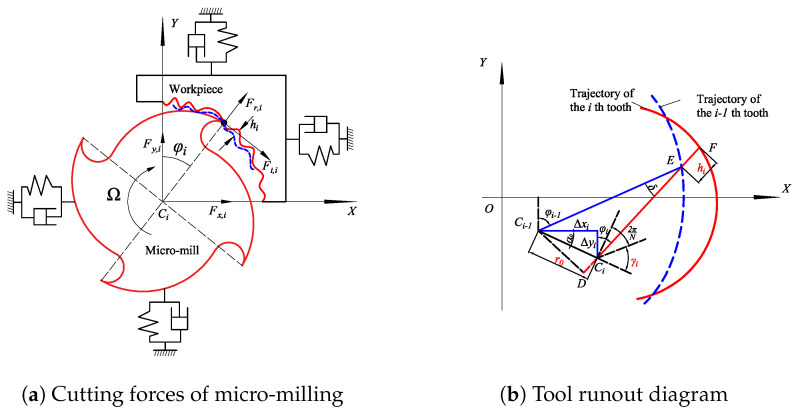
Diagrams of tool runout and cutting forces.

As demonstrated in Figure 6, the cutting thickness $h_{i}$ of the *i*-th tooth is represented by [15]

(21) $$\left\{\begin{array}{c} h_{i}\;=\;EF\;=\;DF-DE\;=\;r_{0}sin\left(\varphi_{i}+\alpha_{0}\right)+R_{D}\left(1-cos\delta \right)\\ DF\;=\;r_{0}sin\left(\varphi_{i}+\alpha_{0}\right)+R_{D}\\ DE\;=\;R_{D}cos\delta \end{array}\right.$$

where $r_{0}$ is the runout distance, $\varphi_{i}$ demotes the position angle of the *i*-th tooth, $R_{D}$ is the tool radius, δ is the angle between two successive teeth, and $\alpha_{0}$ is the runout angle, expressed as

(22) $$\alpha_{0}\;=\;arctan\left(\frac{\Delta y_{i}}{\Delta x_{i}}\right)$$

where $\Delta x_{i}$ and $\Delta y_{i}$ are the runout distances of the micro-mill center in the *X*- and *Y*-directions, respectively.

Substituting the above equation into Equation (21) yields

(23) $$h_{i}\;=\;\Delta x_{i}sin\varphi_{i}+\Delta y_{i}cos\varphi_{i}+R_{D}\left(1-cos\delta \right)$$

where sinδ≈δ and cosδ≈1 are assumed for small angles. Then, the above equation is written as follows:

(24) $$h_{i}\;=\;\Delta x_{i}sin\varphi_{i}+\Delta y_{i}cos\varphi_{i}$$

When the tool center is moved from $C_{i-1}$ to $C_{i}$, $\Delta x_{i}$ and $\Delta y_{i}$ can be written as follows:

(25) $$\Delta_{i}\;=\;\left[\begin{array}{c} \Delta x_{i}\\ \Delta y_{i} \end{array}\right]\;=\;\left[\begin{array}{c} \frac{Nf_{t}}{2\pi }\Delta \varphi_{i}+r_{0}\left[sin\left(\varphi_{i}+\gamma_{i}\right)-sin\left(\varphi_{i}-\Delta \varphi_{i}+\gamma_{i}\right)\right]\\ r_{0}\left[cos\left(\varphi_{i}+\gamma_{i}\right)-cos\left(\varphi_{i}-\Delta \varphi_{i}+\gamma_{i}\right)\right] \end{array}\right]$$

where $f_{t}$ denotes the feed per tooth, $\Delta \varphi_{i}=2\pi /N-\delta$, and *N* denotes the number of teeth. The parameter $h_{i}$ can be calculated by substituting the above equations into Equation (24) [26]:

(26) $$h_{i}\;=\;f_{t}\left(1-\frac{N\delta }{2\pi }\right)sin\varphi_{i}+r_{0}cos\gamma_{i}-r_{0}cos\left(\gamma_{i}+\delta -\frac{2\pi }{N}\right)$$

As depicted in Figure 6, the cutting forces acting on the *i*-th tooth are expressed as

(27) $$\left[\begin{array}{c} F_{x,i}\\ F_{y,i} \end{array}\right]\;=\;\left[\begin{array}{cc} -cos\varphi_{i} & -sin\varphi_{i}\\ sin\varphi_{i} & -cos\varphi_{i} \end{array}\right]\left[\begin{array}{c} F_{t,i}\\ F_{r,i} \end{array}\right]$$

where $F_{x,i}$ and $F_{y,i}$ represent the cutting forces in the X-direction and Y-direction, and $F_{t,i}$ and $F_{r,i}$ denote the cutting forces in the tangential and radial directions and are obtained from [26,27]

(28) $$\left[\begin{array}{c} F_{t,i}\\ F_{r,i} \end{array}\right]\;=\;a_{p}h_{i}\left(t\right)\left[\begin{array}{c} K_{tc}\\ K_{rc} \end{array}\right]+a_{p}\left[\begin{array}{c} K_{te}\\ K_{re} \end{array}\right]$$

where $a_{p}$ represents the axial depth of cut, $K_{tc}$ is the shear force coefficient in the tangential direction, $K_{rc}$ denotes the shear force coefficient in the radial direction, $K_{te}$ indicates the plow force coefficient in the tangential direction, and $K_{re}$ demonstrates the plow force coefficient in the radial direction. In this paper, the cutting force coefficients are modeled as parameters that can be time-varying or fixed to adapt to different cutting situations of the developed model.

The dynamic cutting forces can be formulated by substituting the above equations into Equation (27) and neglecting the second terms [27]:

(29) $$\left[\begin{array}{c} F_{x,i}\\ F_{y,i} \end{array}\right]\;=\;a_{p}\left[\begin{array}{cc} a_{xx,i} & a_{xy,i}\\ a_{yx,i} & a_{yy,i} \end{array}\right]\Delta_{i}$$

where $a_{xx.i}\;=\;-K_{tc}sin\varphi_{i}cos\varphi_{i}-K_{rc}sin^{2}\varphi_{i},a_{xy,i}\;=\;-K_{tc}cos^{2}\varphi_{i}-K_{rc}sin\varphi_{i}cos\varphi_{i},$ $a_{yx,i}\;=\;K_{tc}sin^{2}\varphi_{i}-K_{rc}sin\varphi_{i}cos\varphi_{i},a_{yy,i}\;=\;K_{tc}sin\varphi_{i}cos\varphi_{i}-K_{rc}cos^{2}\varphi_{i}$

## 4. Assembling the Equations

As Figure 1 describes, the cutting forces can be applied to the first node of the simplified spindle–holder–tool assembly. The mathematical model for demonstrating the dynamics of the rotating spindle–holder–tool assembly during cutting can be obtained by adding the matrices in Equations (20) and (29), as follows [6,7,14]:

(30) $$\left[M\right]\left\{\ddot{q}\right\}-\Omega \left[G\right]\left\{\dot{q}\right\}+\left(\left[K\right]-\Omega^{2}\left[M_{c}\right]\right)\left\{q\right\}\;=\;\left[F\right]$$

where $\left\{q\right\}={\left\{q_{1},q_{2}...\right\}}^{T}$, $M\;=\;\sum \left[M^{e}\right],G\;=\;\sum \left[G^{e}\right],K\;=\;\sum \left[K^{e}\right],M_{c}\;=\;\sum \left[M_{c}^{e}\right]$, and

$$\left[F\right]\;=\;\left[\begin{array}{c} \sum_{i\;=\;1}^{N}F_{x,i}\\ \sum_{i\;=\;1}^{N}F_{y,i}\\ \begin{array}{c} 0\\ \vdots \\ 0 \end{array} \end{array}\right]$$

Finally, the obtained dynamic model is characterized by the following:

(1) It includes considerations of the tool runout, centrifugal force, and gyroscopic effect;
(2) It is obtained by combining the FEM and time-domain formulation;
(3) The time-domain, stability lobe, and frequency-domain methods can be applied to analyze the obtained dynamic model.

## 5. Verification and Frequency Analysis

To verify the developed model, experiments on tool-tip FRFs in the X- and Y-directions were conducted using a three-axis CNC Machine (CNC4040F). This machine is equipped with a motorized spindle capable of reaching a maximum rotational speed of 24,000 rpm. The chosen micro-mill for the experiments with two teeth was a tungsten-carbide cutter with a diameter of 0.4 mm. And the results were compared to those of theoretical calculations. Furthermore, micro-milling experiments were carried out. The machined surfaces are presented.

Theoretical calculations and simulation experiments were also employed in this study due to the difficulty of achieving the FRFs of a micro-mill with high rotational speeds. The existing experimental methods for obtaining the FRFs of the spindle–holder–tool system are usually performed at zero rotational speed because it is difficult to apply excitations to rotating tools with high speeds. Tool-tip FRFs were simulated for several high speeds. The material properties and geometric parameters of the spindle–holder–tool system for conditions 1 and 2 are shown in Table 1 and Table 2. The cutting force coefficients for condition 1 and condition 2 are demonstrated in Table 3 and Table 4.

### 5.1. Experimental Analysis of Tool-Tip FRFs

**Figure 7:**
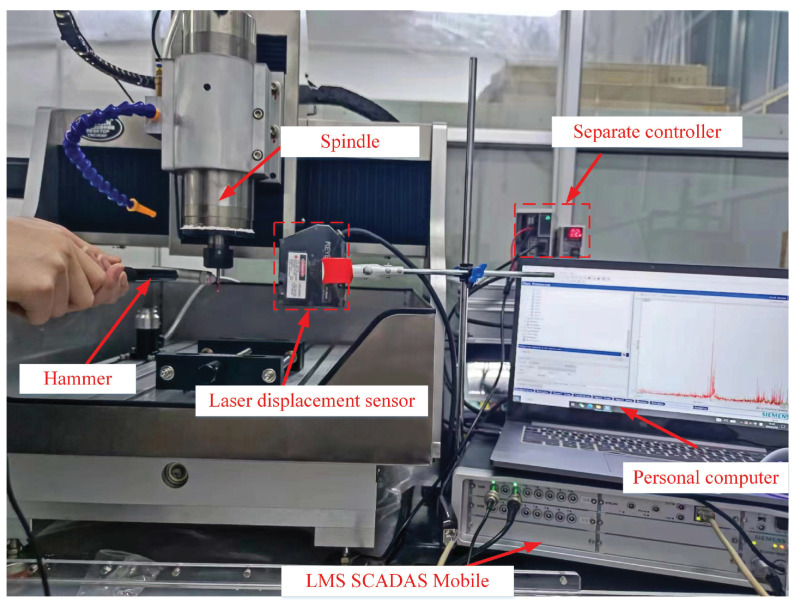
Experimental setup for testing tool-tip FRFs.

Experiments for validating the tool-tip FRFs were carried out. The experimental setup is demonstrated in Figure 7. An impulse hammer (Shiao-SALCO5KE) with a sensitivity of 1 mv/N was used to create impacts on the micro-mill. Vibration displacements of the tool tip were measured using a laser displacement sensor (KEYENCE LK-G80A) with a maximum sampling frequency of 50 K Hz and a sensitivity of 0.13 V/mm. The laser displacement sensor was connected to a multi-channel data acquisition system (LMS SCADAS Moblie) via a separate controller (KEYENCE LK-G3001A), which was also connected to the hammer, to facilitate data acquisition. Considering the fragile nature of the micro-mill, the point of impact was carefully chosen at the shank to prevent any damage.

**Figure 8:**
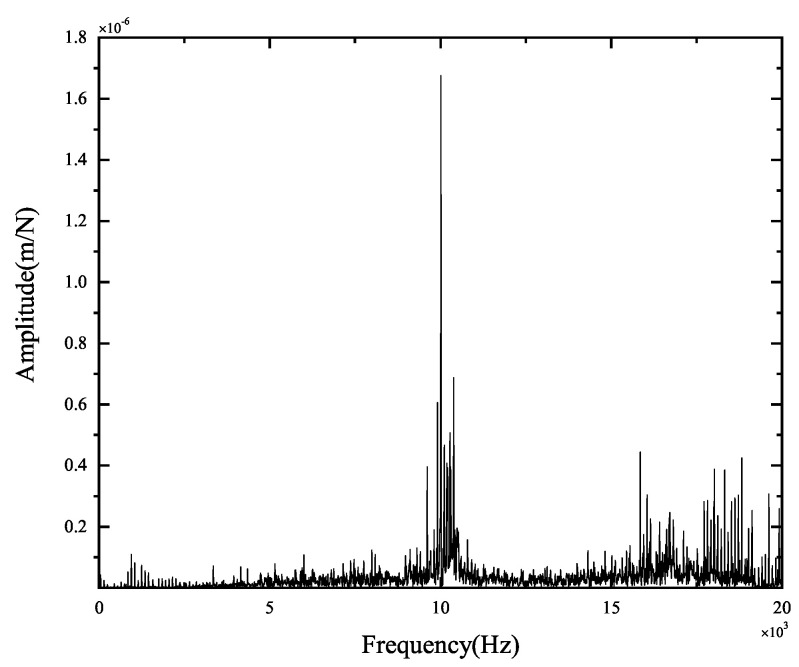
Original experimental FRFs in the X-direction.

**Figure 9:**
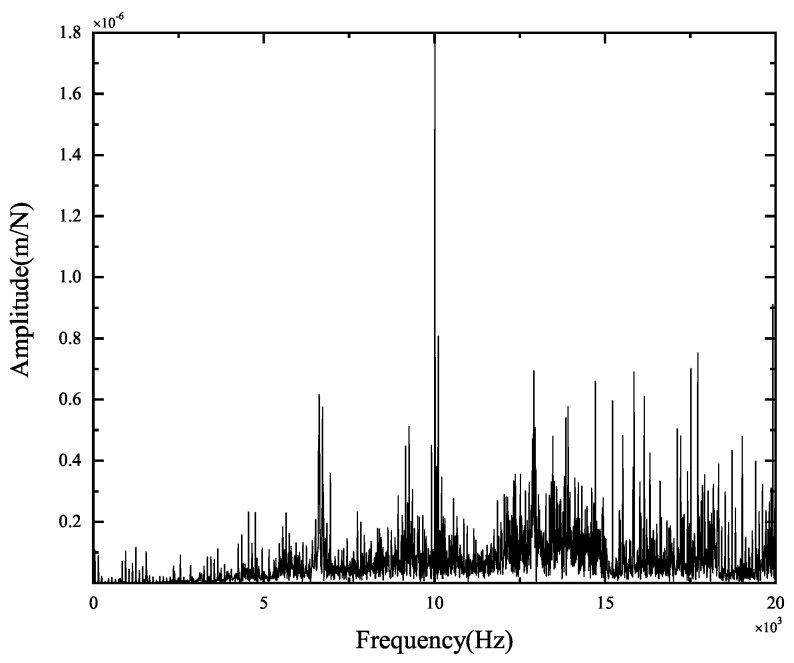
Original experimental FRFs in the Y-direction.

**Figure 10:**
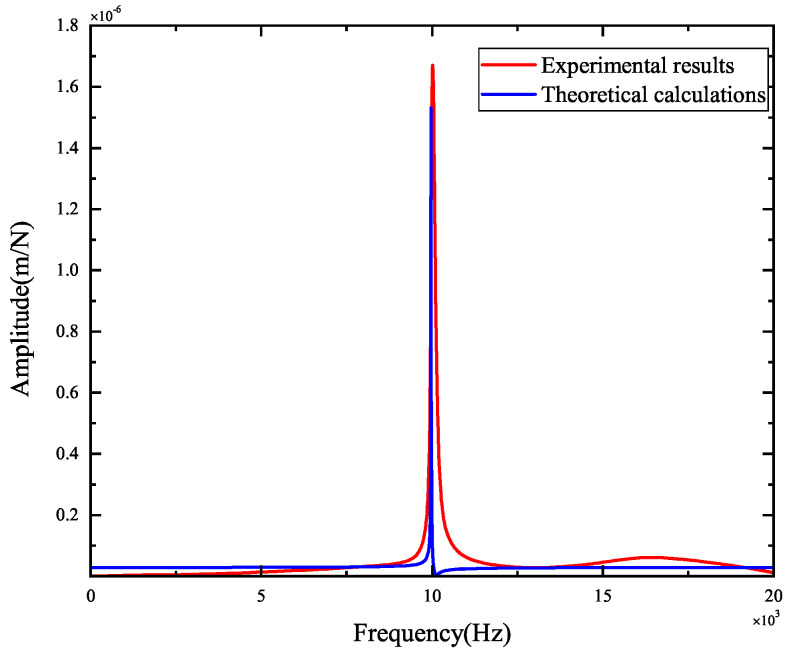
Comparisons of theoretical and experimental FRFs in the X-direction.

**Figure 11:**
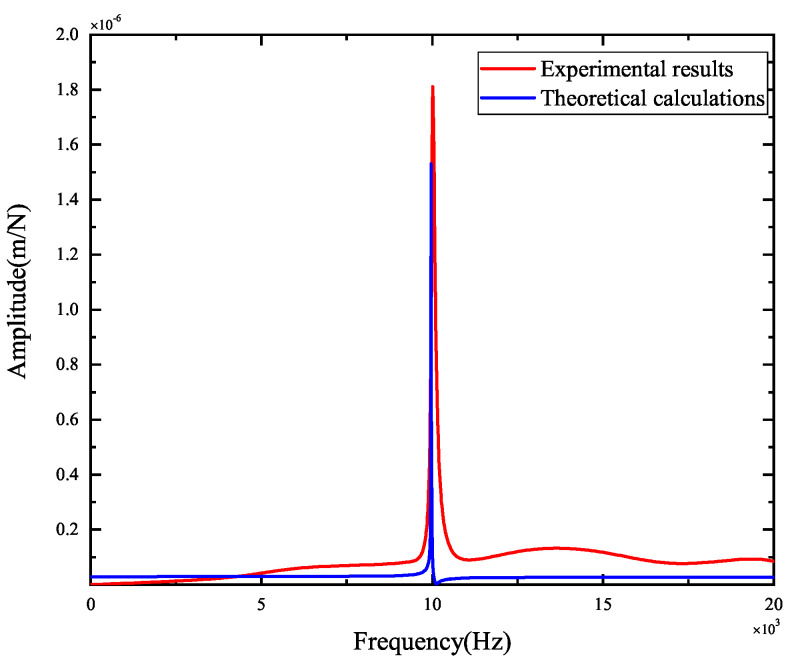
Comparisons of theoretical and experimental FRFs in the Y-direction.

The experimental results of tool-tip FRFs in the X- and Y-directions are illustrated in Figure 8 and Figure 9, respectively. The peak values and the corresponding positions of the frequencies obtained by theoretical and experimental FRFs are compared in Figure 10 and Figure 11. They indicate the agreement between the theoretical calculations and experimental FRFs.

As demonstrated in Figure 8 and Figure 9, the experimental results of FRFs in the X- and Y-directions show that the largest peak value is about $1.8\times 10^{-6}$ m, and the corresponding frequency is about 10,000 Hz, suggesting that the spindle–holder–tool system is sensitive to the corresponding frequencies of disturbances. As shown in Figure 8, the second largest peak value in the X-direction is about $6.87\times 10^{-7}$ m, and the corresponding frequency is about 10,390 Hz, indicating its low sensitivity to the corresponding frequencies of disturbances. Other peak values are less than $6.06\times 10^{-7}$ m, illustrating that the system is not sensitive to the corresponding frequencies of disturbances. As described in Figure 9, the second largest peak value in the Y-direction is about $8.07\times 10^{-7}$ m with a frequency of about 10,010 Hz, and the other peak values are less than $6.60\times 10^{-7}$ m, implying its low sensitivity to the corresponding frequencies of disturbances.

Comparing the theoretical and experimental tool-tip FRFs from Figure 10 and Figure 11 reveals that the maximum error of the largest peak values is $2.82\times 10^{-7}$ m, and the maximum difference between the corresponding frequencies is 39 Hz, indicating that small errors are obtained, and the developed model can be validated.

### 5.2. Frequency Responses at Different Speeds for Condition 1

To analyze tool-tip FRFs at different speeds, theoretical calculations and simulation experiments are employed in this paper.

The material properties and geometric parameters are listed in Table 1 according to [28,29,30] for condition 1.

**Figure 12:**
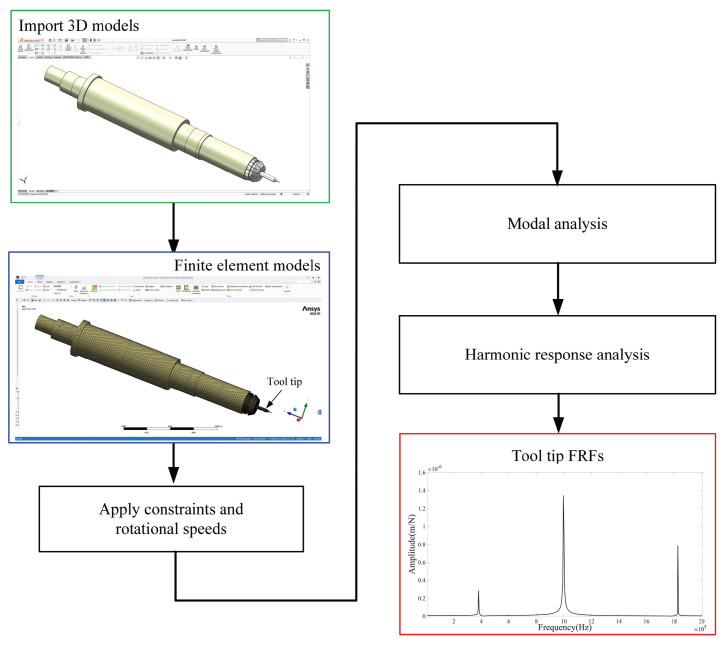
Flowchart of the simulation experiment.

The ANSYS simulation was used to achieve the predicted tool-tip FRFs by modal and harmonic analyses, as shown in Figure 12, where the system models were built using SOLIDWORKS2022.

The MATLAB/SIMULINK simulation calculates the theoretical tool-tip FRFs of the rotating spindle–holder–tool system based on the developed model without tool runout. The presented model was validated by conducting several simulations and matching the frequency responses from MATLAB/SIMULINK 2021a and ANSYS 2022R1 simulations.

**Figure 13:**
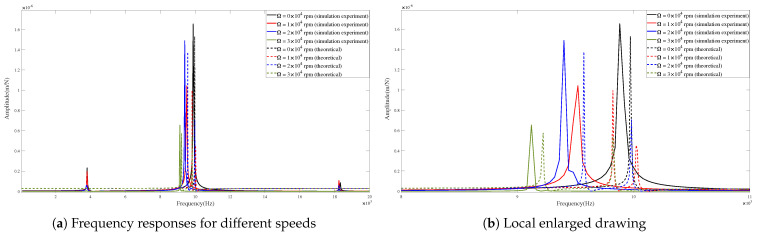
Frequency responses in X-direction for condition 1.

**Figure 14:**
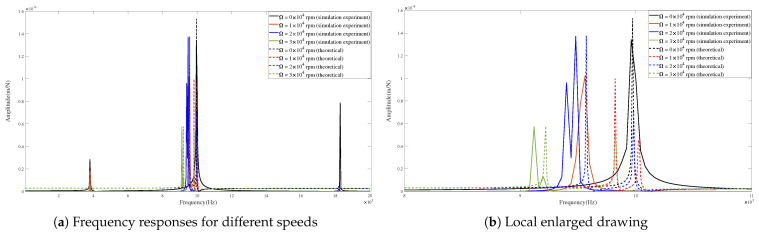
Frequency responses in Y-direction for condition 1.

Figure 13 and Figure 14 show the theoretical and predicted results of tool-tip FRFs in the X- and Y-directions at different rotating speeds.

The FRF peak values are compared in Table 5 and Table 6. The peak values of FRFs obtained by theoretical calculations and captured by the simulation experiments are similar in their key positions and numerical values.

According to Table 5, in the X-direction, the main amplitudes obtained from theory and simulations exhibit minimal differences with small errors (i.e., <0.124 m/N). Furthermore, the positions of the corresponding frequencies at different speeds are very close, with small errors (i.e., <3.162%). According to Table 6, in the Y-direction, the maximum error of peak values is 0.188 m/N, and the maximum error of the corresponding frequencies is 2.730%.

The obtained results indicate an agreement between the theoretical calculations and simulation predictions of FRFs.

Comparing the theoretical and predicted tool-tip FRFs reveals a close agreement, confirming the effectiveness of the presented model. Furthermore, the results suggest that the corresponding frequencies of the peak values decrease with an increase in the rotating speed. This phenomenon can be attributed to the influence of the gyroscopic moment that influences damping. Moreover, the centrifugal force also plays an important role in changing the stiffness. It can be observed that the amplitudes of the dominant mode show a small decline with an increase in the rotating speed. The achieved results agree with those from [31,32,33].

### 5.3. Frequency Responses at Different Speeds for Condition 2

The assumed material properties and geometric parameters for condition 2 are provided in Table 2.

**Figure 15:**
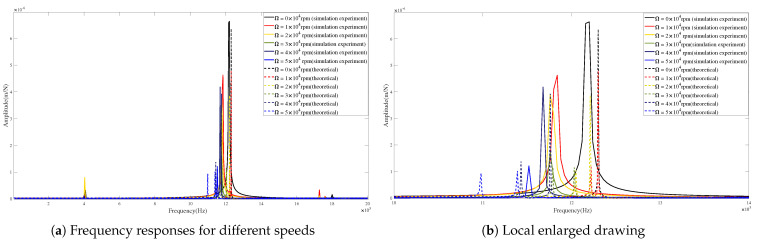
Frequency responses in X-direction for condition 2.

**Figure 16:**
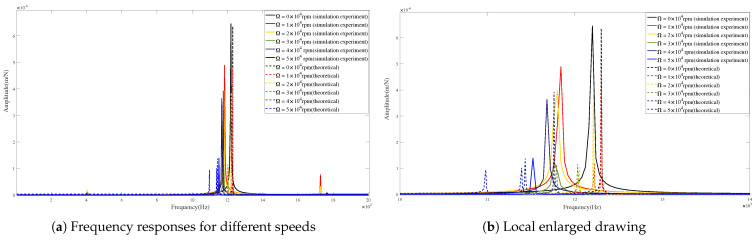
Frequency responses in Y-direction for condition 2.

Figure 15 and Figure 16 show the differences between the theoretical and predicted results of tool-tip FRFs in the X-direction and Y-direction at different rotational speeds. It can be found that the FRF peaks achieved by the theoretical calculations are very close to those obtained from the simulation experiments in critical locations and values.

Table 7 and Table 8 illustrate the variations in amplitude and frequency at different rotational speeds in the X-direction and Y-direction, respectively. It can be seen that the maximum error of amplitudes is 0.582 m/N and the absolute error of frequencies is 3.885% in the X-direction. Meanwhile, in the Y-direction, the analysis reveals a maximum amplitude error of 0.376 m/N and an absolute frequency error of 3.885%. These results show that the theoretical calculations are in agreement with the simulation predictions of tool tip FRFs.

The validity of the proposed model has been demonstrated from the above comparisons. Obviously, the frequencies corresponding to the peaks of tool-tip FRFs decrease with increasing rotational speeds. This phenomenon can be caused by gyroscopic moments and centrifugal forces [31,32].

## 6. Stability Analysis

Several SLDs are compared with the gyroscopic effect and the centrifugal force induced by different rotating speeds. A stability analysis of vibrations is also presented.

This paper mainly focuses on developing a dynamic model for chatter analysis in micro-milling by integrating the effects of the centrifugal force, gyroscopic moment, and tool runout without considering different tool cutting edges and workpiece materials. The effects of different tool cutting edges and workpiece materials are included in the cutting force coefficients.

### 6.1. Stability Analysis of Lobe Diagrams for Condition 1

The parameters of the cutting force coefficients for condition 1 are shown in Table 3 as in [34,35].

**Figure 17:**
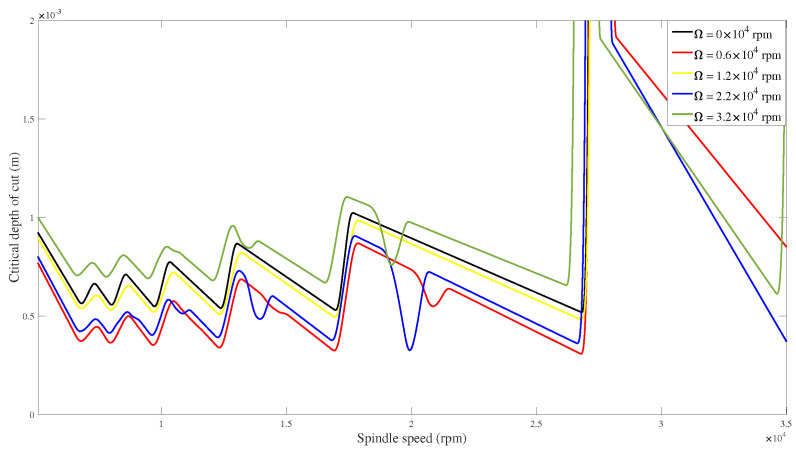
Stability lobe diagrams for condition 1.

Figure 17 demonstrates the simulation results of SLDs and reveals that the critical axial depth of cut is decreased when comparing speeds of 0, $1.2\times 10^{4}$, and $2.2\times 10^{4}$ rpm, indicating a decrease in chatter stability. The results demonstrate that the chatter stability can be roughly decreased by increasing the rotating speed and considering the effects of gyroscopic moments and centrifugal forces.

However, according to Figure 17, the critical axial depth of cut is increased at a speed of $3.2\times 10^{4}$ rpm compared to that at $2.2\times 10^{4}$ rpm. The nonlinear shifts in SLDs suggest the nonlinear effects of rotating speed on SLDs at particular rotating speeds.

The results are consistent with the conclusions from [31,36].

### 6.2. Stability Analysis of Vibrations for Condition 1

**Figure 18:**
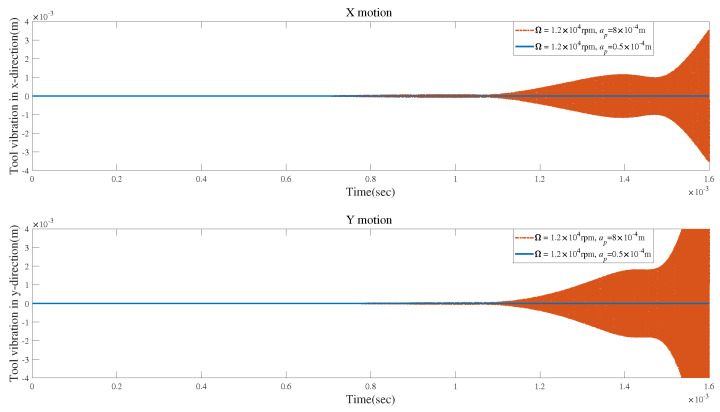
Stable and unstable time responses for condition 1.

As presented in Figure 18, the vibrations are finally stable at an axial depth of cut of $a_{p}\;=\;0.5\times 10^{-4}$ m without tool runout. However, the vibrations display an unstable trend at an axial depth of cut of $a_{p}\;=\;8\times 10^{-4}$ m without tool runout. The stability indicated by the SLDs described in Figure 17 also makes this point.

### 6.3. Stability Analysis of Lobe Diagrams for Condition 2

The parameters of the cutting force coefficients are shown in Table 4 [34,35].

**Figure 19:**
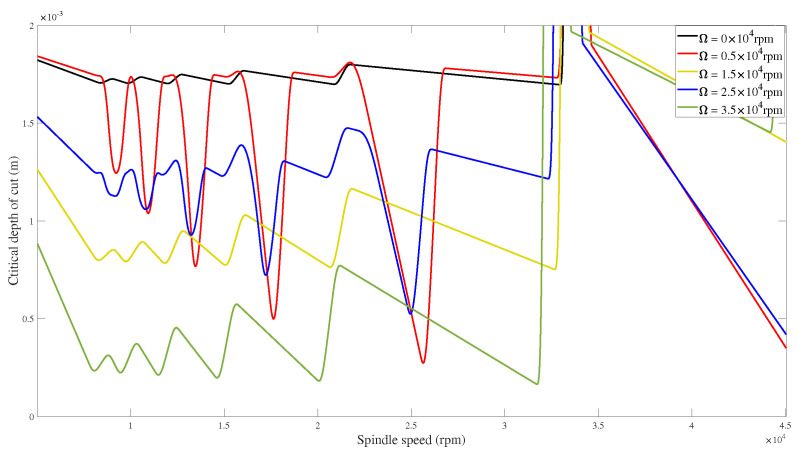
Stability lobe diagrams for condition 2.

Figure 19 shows that the critical depth of cut decreases with an increase in rotating speed for 0, $0.5\times 10^{4}$, $1.5\times 10^{4}$, and $3.5\times 10^{4}$ rpm, indicating a decrease in chatter stability. The critical axial depth of cut exhibits an increase at a speed of $2.5\times 10^{4}$ rpm compared to $1.5\times 10^{4}$ rpm, as depicted in Figure 19. This observation suggests that the rotating speed nonlinearly influences SLDs at specific rotational speeds. This is consistent with previous conclusions [31,36].

### 6.4. Stability Analysis of Vibrations for Condition 2

**Figure 20:**
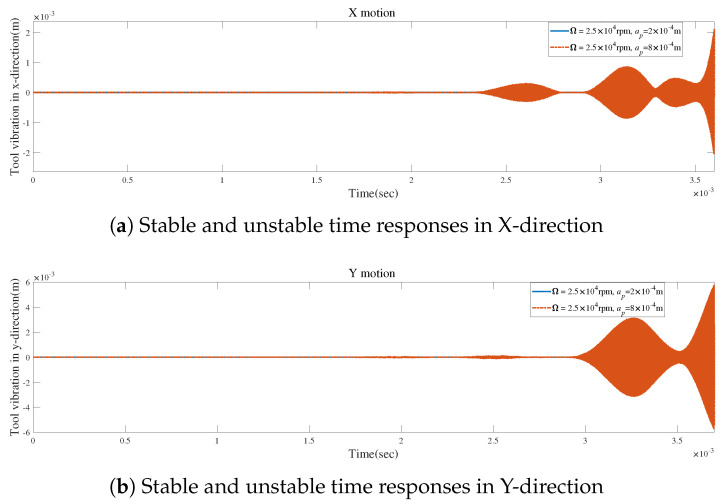
Stable and unstable time responses for condition 2.

Figure 20a,b illustrate that the vibrations reach a stable state when the axial depth of cut is set to $a_{p}\;=\;2\times 10^{-4}$ m without tool runout. The vibrations become unstable at an axial depth of cut of $a_{p}\;=\;8\times 10^{-4}$ m. These results are in agreement with the stability predicted by the SLDs presented in Figure 19.

## 7. Time-Response Analysis

### 7.1. Time-Response Analysis for Condition 1

Simulations of time responses were performed with different parameters to verify the influences of tool runout, the centrifugal force, and the gyroscopic moment on time responses.

The parameters of the cutting force coefficients for condition 1 are listed in Table 3. The parameters of micro-milling are chosen as follows: $f_{t}=2.5\times 10^{-6}$ m, $\Omega =1.2\times 10^{4}$ rpm, $r_{0}\;=\;2\times 10^{-7}$ m, and $r_{i}\;=\;0.001\pi$, where the tool runout continues for $10^{-3}$ s, and the initial value of chatter vibration is $2\times 10^{-7}$ m.

**Figure 21:**
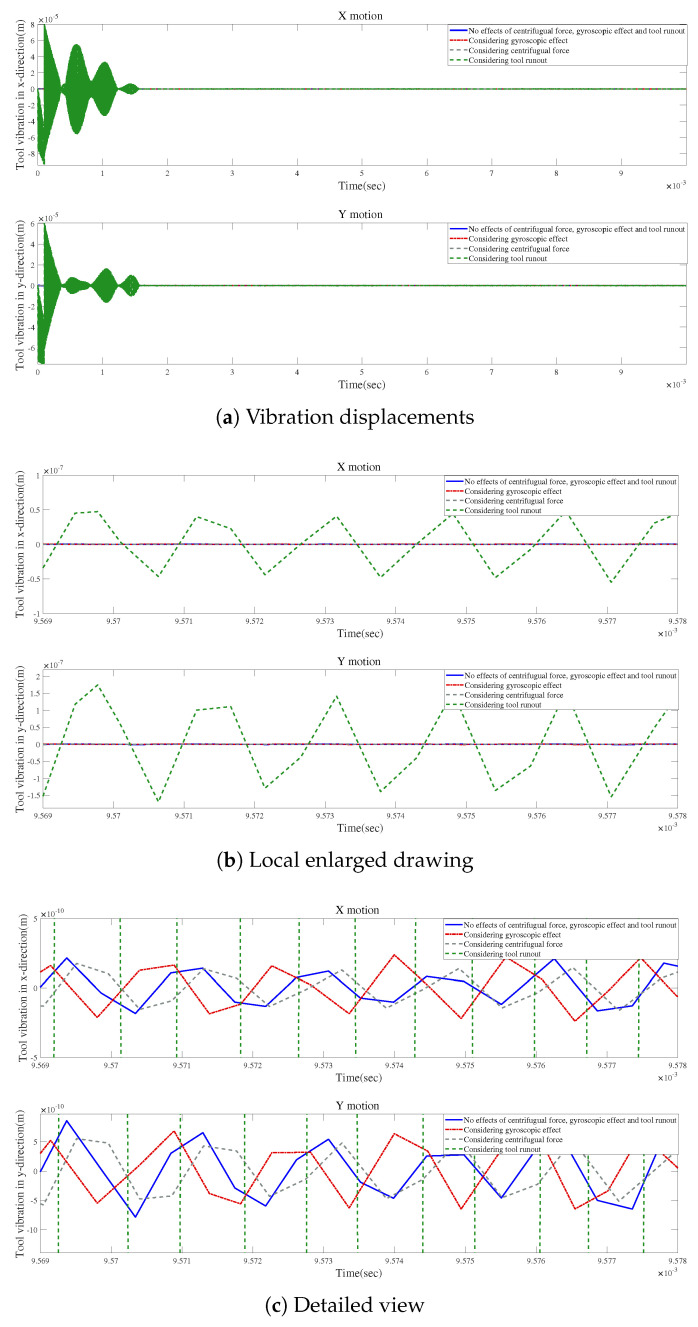
Time responses for condition 1.

According to Figure 21a, small changes in vibration displacements occur when the gyroscopic effect or the centrifugal force is considered without tool runout. However, the vibration displacements can be significantly modified by only considering the influence of tool runout, as shown in Figure 21b. Figure 21c provides a detailed view of the final vibrations, indicating that the final variations in vibration displacement induced by considering the centrifugal force are similar to those generated by considering the gyroscopic effect.

**Figure 22:**
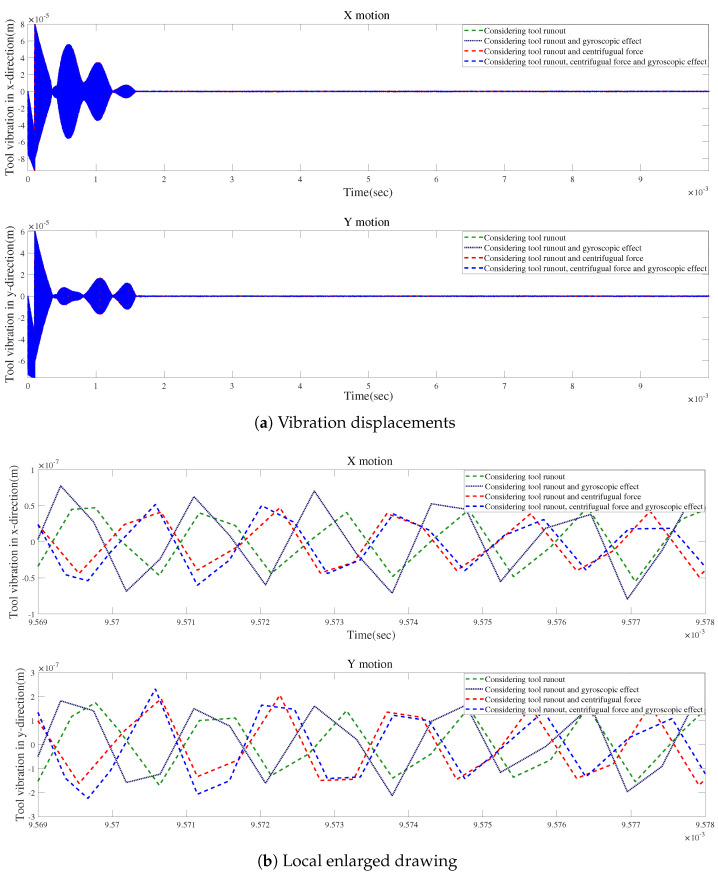
Time responses with tool runout for condition 1.

Figure 22a demonstrates how the factors influence the time-domain responses with tool runout. The largest variation is observed when the gyroscopic effect, the centrifugal force, and tool runout are considered. Figure 22b suggests that the results are similar for the effects of centrifugal force and tool runout. Figure 22 reveals that the three influencing factors can play important roles in the time-domain response, which should not be ignored when modeling the spindle–holder–tool system in micro-milling.

**Figure 23:**
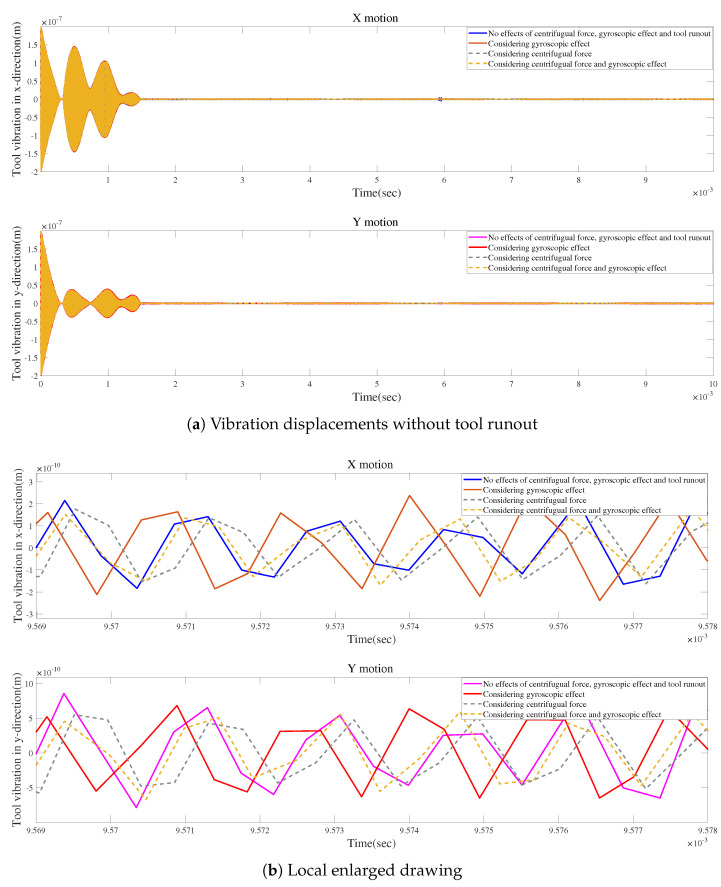
Time responses without tool runout for condition 1.

Figure 23a describes the influences of the gyroscopic moment and centrifugal force on the time-domain responses without tool runout. The highest variation is demonstrated when considering both effects. Figure 23b shows that the two effects may lead to similar variations in vibration displacement.

### 7.2. Time-Response Analysis for Condition 2

Table 4 provides the parameters of the cutting force coefficients. The parameters of micro-milling are selected as follows: $f_{t}=3\times 10^{-6}$ m, $\Omega =3.5\times 10^{4}$ rpm, $r_{0}\;=\;2\times 10^{-7}$ m, and $r_{i}\;=\;0.001\pi$. The tool runout lasts for a duration of $10^{-3}$ s, while the initial value of chatter vibration is $2\times 10^{-7}$ m.

**Figure 24:**
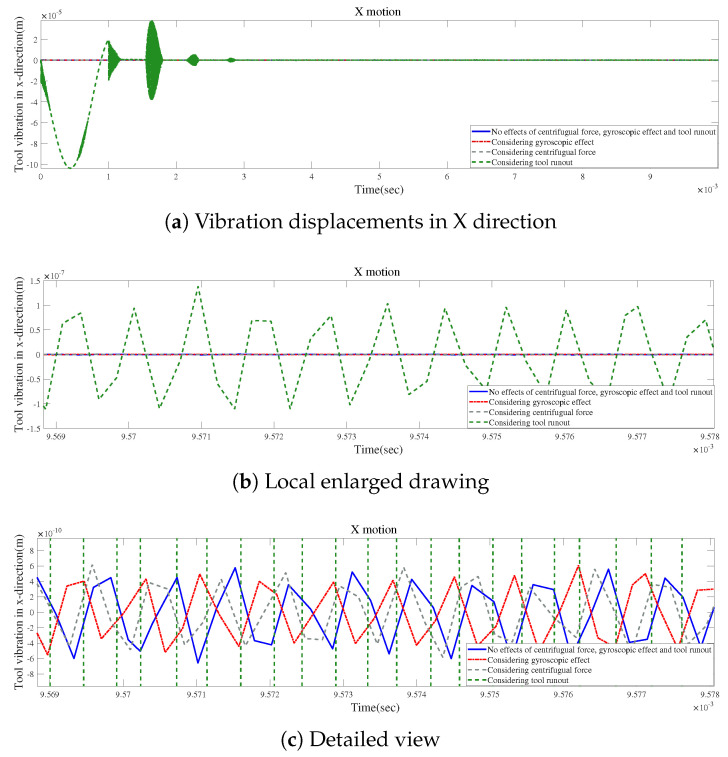
Time responses in X-direction for condition 2.

**Figure 25:**
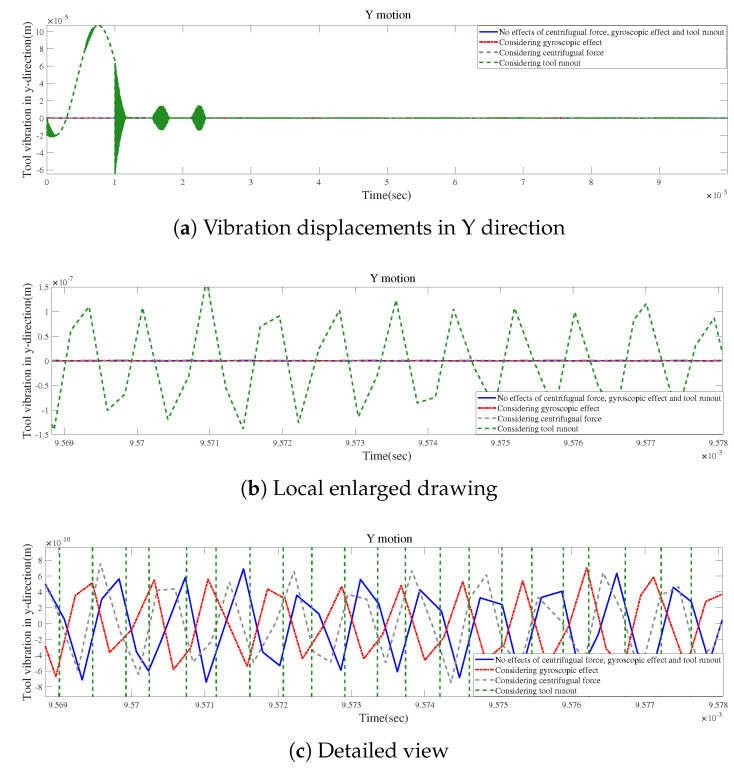
Time responses in Y-direction for condition 2.

According to Figure 24a and Figure 25a, the local enlarged drawings in Figure 24b and Figure 25b, and the detailed views in Figure 24c and Figure 25c, tool runout has a significant impact on the vibration displacement, while the effect of the gyroscopic moment or centrifugal force on vibration displacements is relatively small.

**Figure 26:**
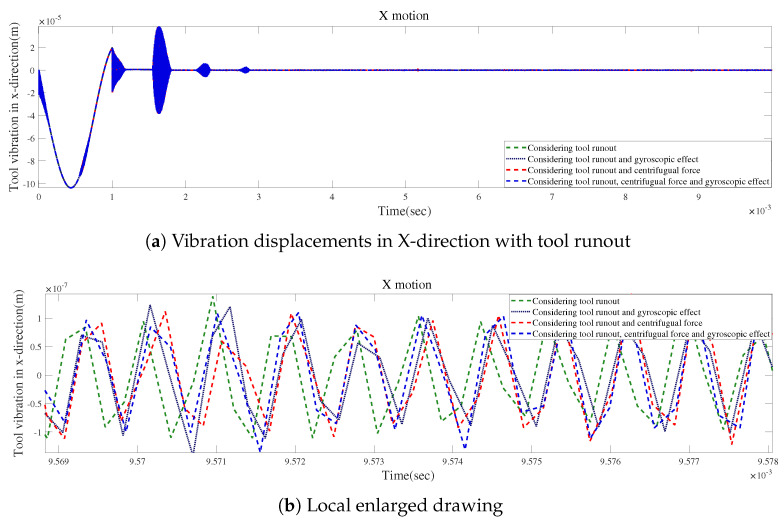
Time responses in X-direction with tool runout for condition 2.

**Figure 27:**
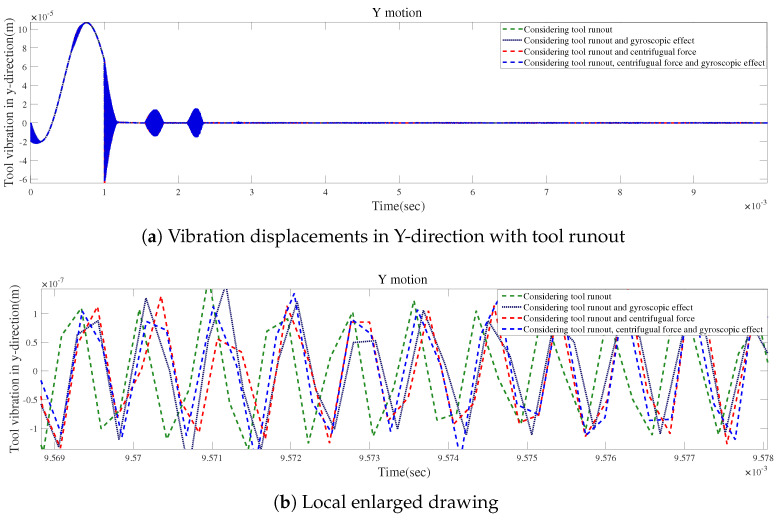
Time responses in Y-direction with tool runout for condition 2.

Figure 26 and Figure 27 demonstrate comparisons of time responses by considering the effects of tool runout, the centrifugal force, and the gyroscopic moment. The results show a similar trend. Figure 26b and Figure 27b reveal that the three influencing factors can contribute to the fluctuations in time-domain responses.

**Figure 28:**
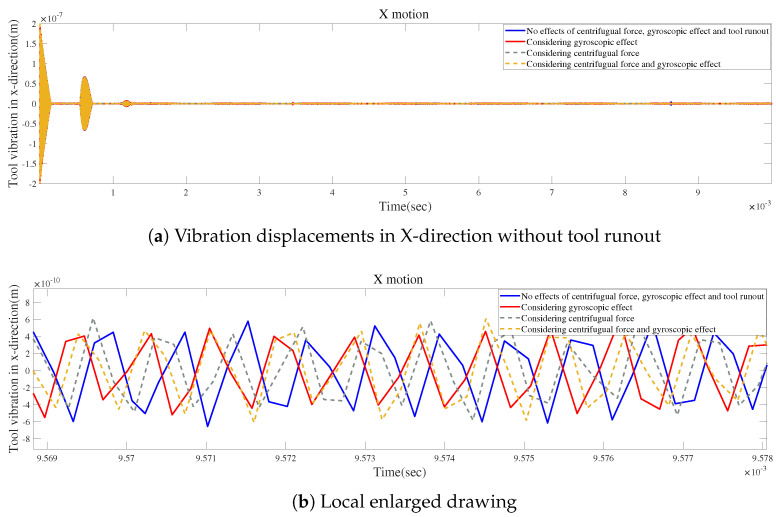
Time responses in X-direction without tool runout for condition 2.

**Figure 29:**
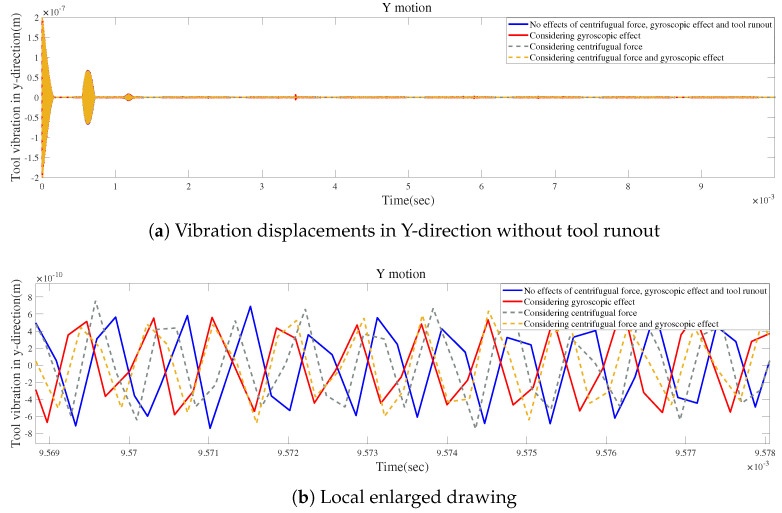
Time responses in Y-direction without tool runout for condition 2.

Figure 28a and Figure 29a show the influences of the gyroscopic moment and centrifugal force on the time-domain responses without tool runout. Figure 28b and Figure 29b illustrate that the two effects may lead to similar variations in vibration displacement.

### 7.3. Effects of Changing Parameters of Tool Runout on Time Responses for Condition 1

The effects of changing the parameters of tool runout on the time response were investigated to analyze their influences on micro-milling.

Different parameters of the runout distance and runout angle result in different time responses of vibrations. Three different conditions are considered: the runout distances for cases A, B, and C are $2\times 10^{-7}$ m, $4\times 10^{-7}$ m, and $2\times 10^{-7}$ m, respectively; the runout angles for cases A, B, and C are $1\times 10^{-3}\pi$, $1\times 10^{-3}\pi$, and $3\times 10^{-3}\pi$, respectively. The duration times for cases A, B, and C range from 0 to $0.1\times 10^{-3}$ s. The parameters of micro-milling are the same as in condition 1.

**Figure 30:**
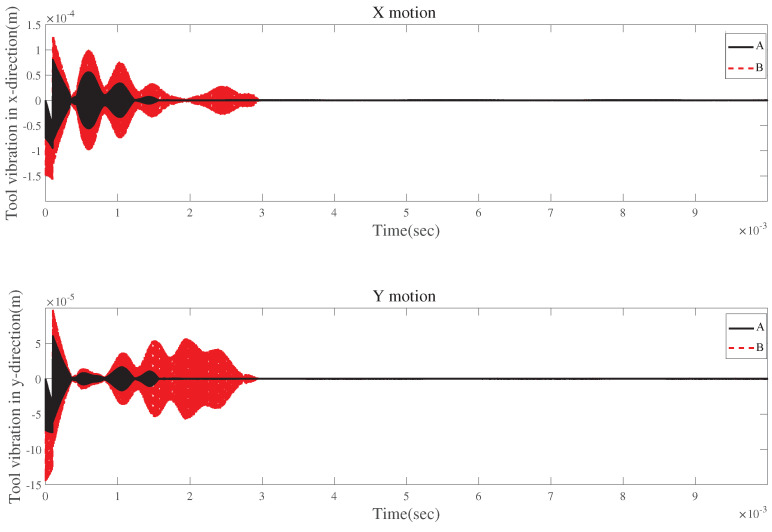
Time responses for cases A and B with tool runout.

**Figure 31:**
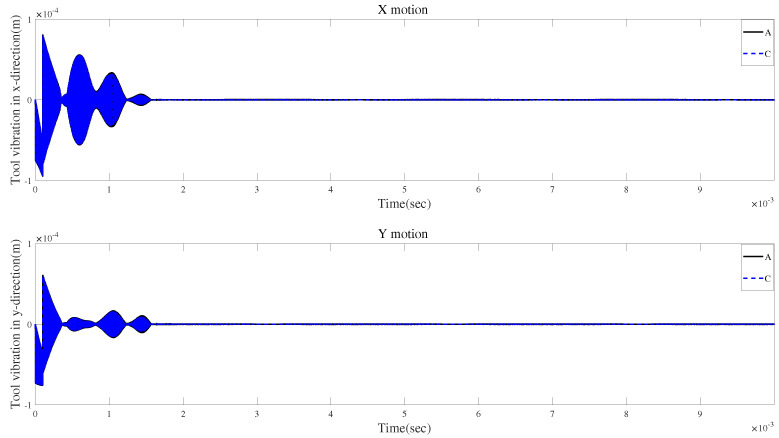
Time responses for cases A and C with tool runout.

The results are demonstrated in Figure 30 and Figure 31. Figure 30 indicates that a larger runout distance can lead to larger vibrations, and it will take a longer time for the cutting system to stabilize the vibrations. Figure 31 suggests that the impact of a larger runout angle on the time response is relatively small.

### 7.4. Effects of Changing Parameters of Cutting Force Coefficients on Time Responses

The cutting force coefficients can be time-varying or fixed due to different cutting situations. Two commonly used methods for the identification of cutting force coefficients are linear regression analysis [37] and the equilibrium-optimizer-based method [38]. To investigate the effects of changing the parameters of cutting force coefficients on time responses, four simulations were carried out with assumed time-varying cutting force coefficients, which were used to represent the identified ones.

Two different conditions are considered. For condition 1, for which the material properties and geometric parameters are presented in Table 1, the initial values of the chatter vibrations are $2\times 10^{-7}$ m, $f_{t}=2.5\times 10^{-6}$ m, $a_{p}=0.02$ mm, $\Omega =2.2\times 10^{4}$ rpm, $r_{0}\;=\;2\times 10^{-7}$ m, and $r_{i}\;=\;0.001\pi$. The tool runout lasts for a duration of $10^{-3}$ s. Two cases, case A and case B, are investigated. In case A, the time-varying cutting force coefficients are 1.06×|sin(t)|× the fixed cutting force coefficients presented in Table 3, with *t* as the simulation time. In case B, the time-varying cutting force coefficients are 0.94×|sin(t)|× the fixed cutting force coefficients presented in Table 3.

For condition 2, for which the material properties and geometric parameters are presented in Table 2, $f_{t}=2.0\times 10^{-6}$ m, $a_{p}=0.2$ mm, and $\Omega =1.5\times 10^{4}$ rpm, and the other parameters of cutting are the same as those of condition 1. Two cases, case C and case D, are explored. Cases C and D, respectively, denote time-varying cutting force coefficients that are 1.05×|sin(t)|× the fixed cutting force coefficients presented in Table 4 and 0.95×|sin(t)|× the fixed cutting force coefficients presented in Table 4.

**Figure 32:**
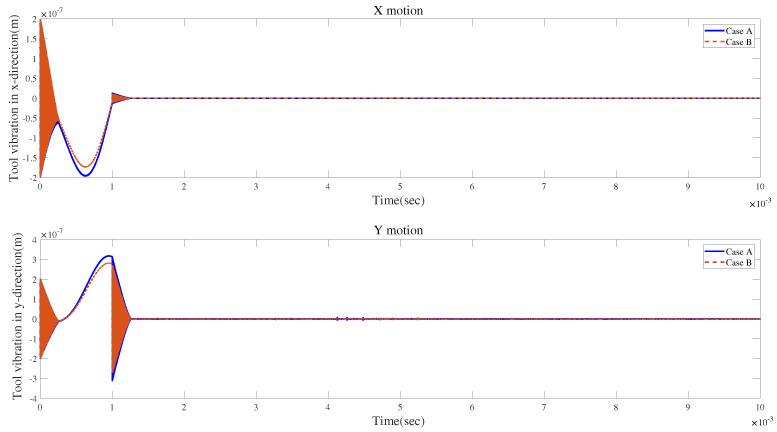
Time responses for cases A and B with time-varying cutting force coefficients.

**Figure 33:**
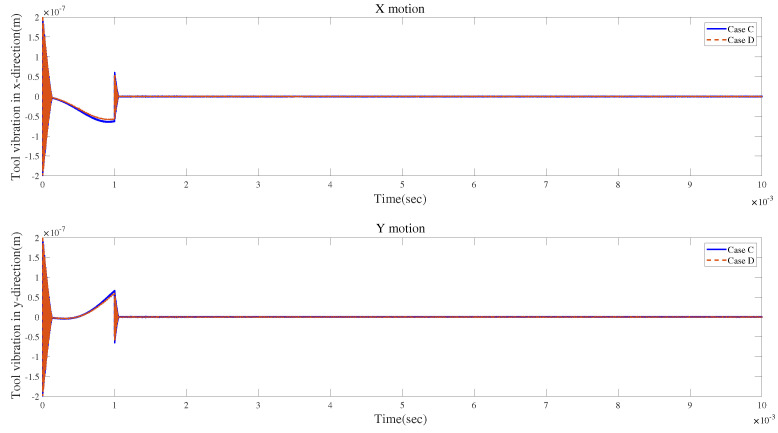
Time responses for cases C and D with time-varying cutting force coefficients.

The time responses with time-varying cutting force coefficients are shown in Figure 32 and Figure 33. The figures indicate that different cutting force coefficients, which may be time-varying due to different cutting situations, can lead to differences in the time responses of vibrations.

### 7.5. Experiments for Micro-Milling

**Figure 34:**
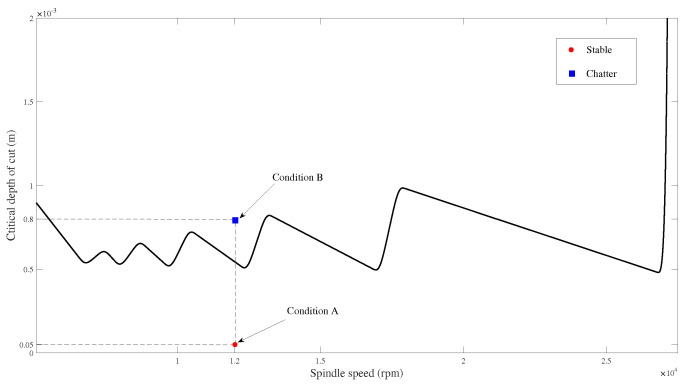
The stable and unstable regions predicted by SLD.

**Figure 35:**
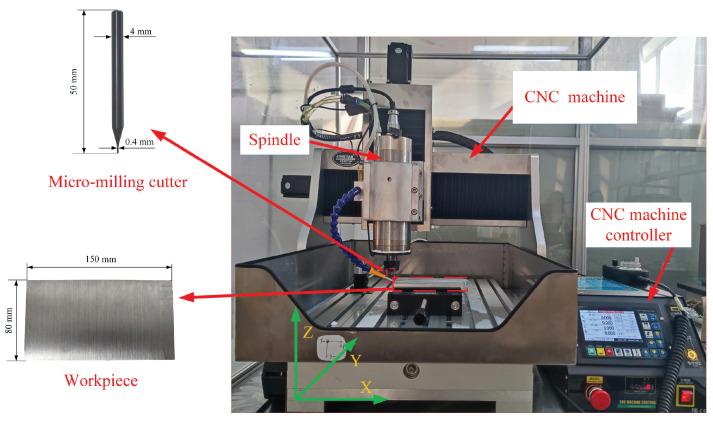
Experimental setup for micro-milling.

To verify the effectiveness of the calculated SLD in Figure 34, which is used to predict the stable and unstable regions between different spindle speeds and depths of cut, micro-milling experiments were carried out. Figure 35 illustrates the experimental setup for micro-milling. A micro-mill with a diameter of 0.4 mm and two teeth was used for micro-milling. The workpiece material was aluminum 6061 with a size of 150 mm × 80 mm × 15 mm. During the experiment, the spindle speed was set to $\Omega \;=\;1.2\times 10^{4}$ rpm, and the feed was maintained at 10 mm per minute.

**Figure 36:**
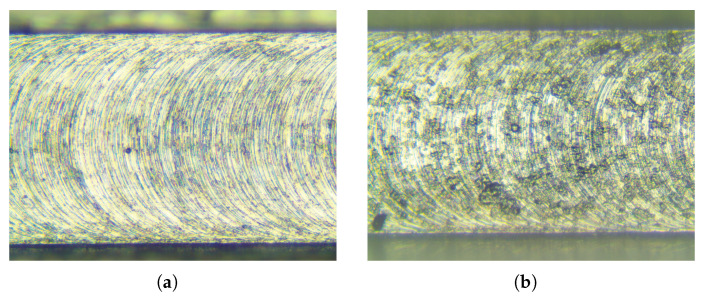
Machined surfaces of workpiece. (**a**) Machined surface for cutting condition A ($\Omega \;=\;1.2\times 10^{4}$ rpm, $a_{p}\;=\;0.05$ mm). (**b**) Machined surface for cutting condition B ($\Omega \;=\;1.2\times 10^{4}$ rpm, $a_{p}\;=\;0.8$ mm).

As shown by the SLD in Figure 34, point A, which represents the condition with a rotational speed $\Omega \;=\;1.2\times 10^{4}$ rpm and an axial depth of cut $a_{p}\;=\;0.05$ mm, and point B, which represents the condition with a rotational speed $\Omega \;=\;1.2\times 10^{4}$ rpm and an axial depth of cut $a_{p}\;=\;0.8$ mm, are selected to present the stable and unstable cutting conditions for micro-milling. Figure 36 displays the machined surfaces of a workpiece under different selected cutting conditions. The pictures of machined surfaces were captured by using an optical microscope (Aosvi U3CMO5). For a rotational speed $\Omega \;=\;1.2\times 10^{4}$ rpm and an axial depth of cut $a_{p}\;=\;0.05$ mm, the machined surface is shown in Figure 36a. The results reveal a regular distribution of machined waves with distinct separation between neighboring processes, indicating a stable cutting condition in micro-milling. For a rotational speed $\Omega \;=\;1.2\times 10^{4}$ rpm and an axial depth of cut $a_{p}\;=\;0.8$ mm, the machined surface is shown in Figure 36b. Conversely, irregular machined waves were observed on the machined surface in this condition, illustrating an unstable cutting condition.

According to the above analyses of the experiments and the calculated SLD, a satisfactory coincidence between theoretical and experimental results is achieved. Subsequently, stable cutting conditions of different spindle speeds and depths of cut for micro-milling can be predicted and applied for actual cutting, preventing unstable cutting conditions and obtaining the required precision of machined surfaces.

## 8. Conclusions

In this study, a mathematical model for describing the dynamics of the regenerative chatter behavior of micro-milling was developed by using the FEM and time-domain formulation and considering tool runout, the centrifugal force, and the gyroscopic moment. The developed model was verified by comparing the tool-tip FRFs obtained by theoretical calculations and those obtained via a simulation test. The SLDs and time responses were analyzed, and the following points are summarized:

1. In this paper, the development of an integrated modeling for chatter analysis in micro-milling is presented by integrating the effects of the centrifugal force, the gyroscopic moment and tool runout.
2. Comparing the theoretical and experimental tool-tip FRFs reveals that the maximum error of the largest peak values is $2.82\times 10^{-7}$ m, and the maximum difference of corresponding frequencies is 39 Hz, indicating that small errors are obtained, and the developed model can be validated. Moreover, the stable and unstable cutting conditions for micro-milling were predicted by the SLD and verified by cutting experiments. The comparison between the actual cutting conditions for micro-milling experiments and those predicted by the SLD shows their agreement, illustrating the effectiveness of the developed model.
3. The theoretical results of tool-tip FRFs calculated from the developed model with high speeds are approximately consistent with the simulated results, indicating the validity of the developed equations.
4. The results of SLDs suggest that the critical axial depth of cut decreases with an increase in rotating speed within a specific range. Furthermore, the nonlinear shifts in SLDs imply nonlinear influences of the centrifugal force and nonlinear impacts of the gyroscopic moment on SLD.
5. The highest variations in the time-domain response are observed when the effects of tool runout, the centrifugal force, and the gyroscopic moment are included.
6. The results show that the developed model can be used to demonstrate the influences of tool runout, the centrifugal force, and the gyroscopic effect on regenerative chatter behavior, including tool-tip FRFs, SLDs, and time responses.
7. The chatter control method will be investigated in the future.

## Figures and Tables

**Table 1:** Material properties and geometric parameters of spindle–holder–tool system for condition 1.

Parameters	Spindle	Holder	Tool	Cutter
Elastic modulus *E* (Pa)	$2.1\times 10^{11}$	$2.1\times 10^{11}$	$6.08\times 10^{11}$	$6.08\times 10^{11}$
Poisson ratio μ	0.3	0.3	0.23	0.23
Density $\rho \;(\mathrm{kg}/m^{3})$	7900	7850	14,000	14,000
Length L(m)	0.198	0.031	0.0136	0.003
Radius R(m)	0.0135	0.0105	0.002	0.0002

**Table 2:** Material properties and geometric parameters of spindle–holder–tool system for condition 2.

Parameters	Spindle	Holder	Tool	Cutter
Elastic modulus *E* (Pa)	$2.1\times 10^{11}$	$2.1\times 10^{11}$	$2.1\times 10^{11}$	$6.3\times 10^{11}$
Poisson ratio μ	0.3	0.3	0.3	0.25
Density $\rho \;(\mathrm{kg}/m^{3})$	7850	7850	7850	8100
Length L(m)	0.219	0.031	0.0014	0.002
Radius R(m)	0.0125	0.0105	0.0015	0.00015

**Table 3:** Cutting force coefficients for condition 1.

Number of Teeth	Ktc	Krc	Kte	Kre
2	$3.6\times 10^{9}\;N/m^{2}$	$3.5\times 10^{9}\;N/m^{2}$	$1.3\times 10^{4}\;N/m$	$1\times 10^{4}\;N/m$

**Table 4:** Cutting force coefficients for condition 2.

Number of Teeth	Ktc	Krc	Kte	Kre
2	$8.5\times 10^{8}\;N/m^{2}$	$8\times 10^{8}\;N/m^{2}$	$3.3\times 10^{4}\;N/m$	$3\times 10^{4}\;N/m$

**Table 5:** Comparisons of peak values in X-direction for condition 1.

Rotation Speed (rpm)	Amplitude ($10^{-6}\cdot m/N$)		Frequency (Hz)	
	Theoretical	Simulation	Theoretical	Simulation
0	1.530	1.654	9971	9880
10,000	0.997	1.042	9821	9520
20,000	1.375	1.488	9571	9400
30,000	0.575	0.654	9221	9120

**Table 6:** Comparisons of peak values in Y-direction for condition 1.

Rotation Speed (rpm)	Amplitude ($10^{-6}\cdot m/N$)		Frequency (Hz)	
	Theoretical	Simulation	Theoretical	Simulation
0	1.530	1.342	9971	9960
10,000	0.997	1.020	9821	9560
20,000	1.375	1.371	9571	9480
30,000	0.575	0.572	9221	9120

**Table 7:** Comparisons of peak values in X-direction for condition 2.

Rotation Speed (rpm)	Amplitude ($10^{-6}\cdot m/N$)		Frequency (Hz)	
	Theoretical	Simulation	Theoretical	Simulation
0	6.362	6.640	12,301	12,200
10,000	4.805	4.628	12,300	11,840
20,000	3.864	3.900	12,211	11,760
30,000	1.150	1.732	11,780	11,745
40,000	3.925	4.184	11,760	11,680
50,000	1.011	1.209	11,390	11,520

**Table 8:** Comparisons of peak values in Y-direction for condition 2.

Rotation Speed (rpm)	Amplitude ($10^{-6}\cdot m/N$)		Frequency (Hz)	
	Theoretical	Simulation	Theoretical	Simulation
0	6.362	6.459	12,301	12,200
10,000	4.805	4.896	12,300	11,840
20,000	3.864	3.900	12,211	11,800
30,000	1.150	1.254	11,791	11,760
40,000	3.925	3.639	11,760	11,680
50,000	1.011	1.387	11,390	11,520

## Data Availability

Data is contained within the article.
